# Connecting Peptide Physicochemical and Antimicrobial Properties by a Rational Prediction Model

**DOI:** 10.1371/journal.pone.0016968

**Published:** 2011-02-09

**Authors:** Marc Torrent, David Andreu, Victòria M. Nogués, Ester Boix

**Affiliations:** 1 Department of Biochemistry and Molecular Biology, Biosciences Faculty, Universitat Autònoma de Barcelona, Cerdanyola del Vallès, Spain; 2 Department of Experimental and Health Sciences, Barcelona Biomedical Research Park (PRBB), Pompeu Fabra University, Barcelona, Spain; Charité, Campus Benjamin Franklin, Germany

## Abstract

The increasing rate in antibiotic-resistant bacterial strains has become an imperative health issue. Thus, pharmaceutical industries have focussed their efforts to find new potent, non-toxic compounds to treat bacterial infections. Antimicrobial peptides (AMPs) are promising candidates in the fight against antibiotic-resistant pathogens due to their low toxicity, broad range of activity and unspecific mechanism of action. In this context, bioinformatics' strategies can inspire the design of new peptide leads with enhanced activity. Here, we describe an artificial neural network approach, based on the AMP's physicochemical characteristics, that is able not only to identify active peptides but also to assess its antimicrobial potency. The physicochemical properties considered are directly derived from the peptide sequence and comprise a complete set of parameters that accurately describe AMPs. Most interesting, the results obtained dovetail with a model for the AMP's mechanism of action that takes into account new concepts such as peptide aggregation. Moreover, this classification system displays high accuracy and is well correlated with the experimentally reported data. All together, these results suggest that the physicochemical properties of AMPs determine its action. In addition, we conclude that sequence derived parameters are enough to characterize antimicrobial peptides.

## Introduction

Antimicrobial peptides (AMPs) are molecules found in all biological kingdoms responsible for the fight against microbial infections in the first steps of the immunological response [1]. New strategies developed by bacteria and other microorganisms to evade classical antibiotics have urged the pharmaceutical industry to develop new drugs in order to wipe out these resistant microorganisms [2]. In particular, AMPs have been proposed as promising candidates against these pathogens [3], [4]. Although AMPs show a low potency when compared with the small bioactive drugs used at present, they offer counterweigh advantages such as broad range of activity and low toxicity and are less prone to give rise to resistant strains [5], [6], [7]. However, AMPs may present some drawbacks such as serum instability [8], degradation by proteases [9] and high production costs in the case of large polypeptides.

Hence, some *in silico* methods have been developed to find AMPs with potential therapeutic application. Several algorithms take advantage of data mining and high-throughput screening techniques and apply vector-like analysis to scan protein and peptide sequences [10], [11]. Other bioinformatics' strategies include supervised learning techniques, such as artificial neural networks (ANN) or support vector machines (SVM), in order to evaluate easily and reliably a great amount of complex data [12], [13]. In fact, most attempts have been centred in the prediction of highly active peptides using quantitative structure-activity relationships (QSAR) descriptors together with ANN [14], [15], [16], linear discriminant [17] or principal component analysis [18]. These systems use mainly 3D-QSAR descriptors to detail the antimicrobial properties of peptides. Recently, a QSAR-based ANN system was experimentally validated using SPOT high-throughput peptide synthesis, showing that this methodology can accomplish a reliable prediction by means of conventional and “inductive” QSAR descriptors [19]. However, the datasets used contained only peptides with fixed length and the leads found were only populated in few amino acids (W, R and K and, more limitedly, L, V and I). Although AMPs are actually enriched in these residues, a wide diversity in the amino acid content can be found in natural AMPs [20].

Despite the inherent complexity in designing a prediction system only by means of computational chemistry, the recent methods mentioned above have made a remarkable advance. Hence, the combined use of bioinformatics and experimental screening techniques will be essential for the discovery and refinement of new AMPs [21].

We report here an ANN based method that is able to correlate a complete set of sequence-derived physicochemical properties with antimicrobial activity. The most pioneering feature in this method is the ability to translate the observed results in a model of action for AMPs taking into account some new concepts such as peptide aggregation. Additionally, we conclude that the amino acid sequence provides us with sufficient information to accurately predict antimicrobial peptides.

## Results and Discussion

### Physicochemical descriptors of antimicrobial peptides

**Figure 1:**
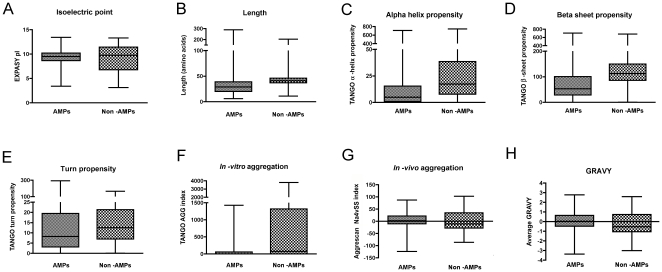
Statistical distribution of peptide physicochemical properties. Each panel corresponds to one parameter: (A) isoelectric point, (B) length, (C) α-helix propensity, (D) β-sheet propensity, (E) turn propensity, (F) *in vitro* aggregation, (G) *in vivo* aggregation and (H) hydrophobicity. The left box in each panel stands for the AMPs dataset and the right box to the non-AMPs dataset. Propensity values for *in vitro* aggregation and secondary structure prediction were computed using the TANGO software [26]. *In vivo* aggregation propensity was computed using AGGRESCAN software (The Na4vSS values computed represent the average aggregation propensity over the entire sequence divided by the number of residues and multiplied by 100) [27]. The isoelectric point (pI) was computed using the Expasy reference values and the peptide hydrophobic mean character using the GRAVY scale.

In order to characterize the physicochemical properties of AMPs, we have selected eight parameters: isoelectric point (pI), peptide length, α-helix, β-sheet and turn structure propensity, *in vivo* and *in vitro* aggregation propensity and hydrophobicity. We have first evaluated these descriptors in antimicrobial and non-antimicrobial peptides to test its suitability to act as classifiers (See the *Methods* section for a complete description on the peptide datasets). The statistical distribution for each parameter is illustrated in Figure 1.

It can be seen in Figure 1A that AMPs and non-AMPs have similar average isoelectric points of 9.26 and 9.20, respectively. However, the variances observed for both groups are significantly different (p-value <0.0001), being the data dispersion much greater for non-AMPs. Thus, variance analysis suggest that a high positive net charge is required for AMPs whereas it does not represent a distinctive feature in non-AMPs, probably due to the diverse functions exerted by these peptides.

Similar results are observed for peptide length (Figure 1B). Non-AMPs tend to be larger than AMPs but no significant differences can be found in the mean value though variances in both groups differ significantly (p-value <0.0001). The length parameter can contribute to the antimicrobial mechanism of action by modulating the peptide insertion into the membrane [22]. It has also been described to be an important parameter for the *de-novo* generation of antimicrobial peptides [23].

**Figure 2:**
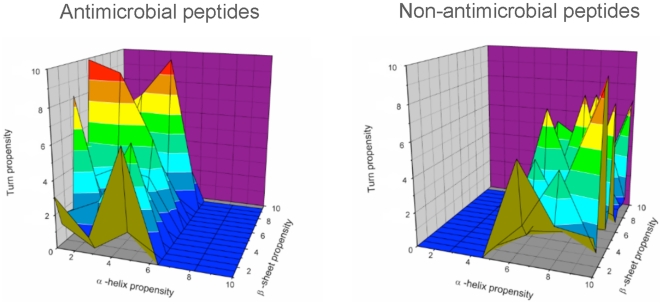
Structure propensity plots, showing α-helix, β-sheet and turn propensity for non-AMPs (top) and AMPs (bottom). The propensity values were calculated using the TANGO software [26] and were rescaled from 1 to 10 to help visual inspection.

Remarkable differences have been found in the analysis of the structural parameters of these two groups (Figures 1C, 1D, 1E). In this case, it can be observed that AMPs tend to be random coil in solution, with a low tendency to present any defined structure. In contrast, non-AMPs display a high proclivity to adopt α-helix or β-sheet structure (Figure 2). These results are consistent with the experimental data reported [22] where it has been described that most AMPs have no structure in solution but acquire a defined secondary structure upon membrane interaction.

We have also studied the aggregation propensity for both groups, as it could be an important modulator of the peptide function. Aggregation in solution has to be clearly distinguished from the capacity of many antimicrobial peptides to form aggregates upon interaction with cell membranes, a step required for the AMPs mode of action (e.g. in the “*carpet –like*” mechanism [22]). Besides, peptide aggregation on the bacteria surface has been observed in some cell-agglutinating AMPs [24], [25]. This agglutinating activity could help bacteria clearance in the body at the infection focus [3].

It has been described that TANGO is a good predictor of aggregation in solution, as it uses sequence-derived structural parameters to forecast aggregation [26]. On the other hand, AGGRESCAN is a good analyst of aggregation in bacteria, as it has been developed in an *E. coli* system [27], [28]. We have used TANGO software to predict *in vitro* (or “*in solution*”) aggregation, whereas AGGRESCAN has been used to predict *in vivo* aggregation. As we can see in Figures 1F and 1G, AMPs display a low *in vitro* aggregation propensity when compared with non-AMPs while the *in vivo* aggregation parameter is considerably higher for AMPs. Additionally, great dispersion in the *in vitro* aggregation propensity has also been observed for non-AMPs.

**Figure 3:**
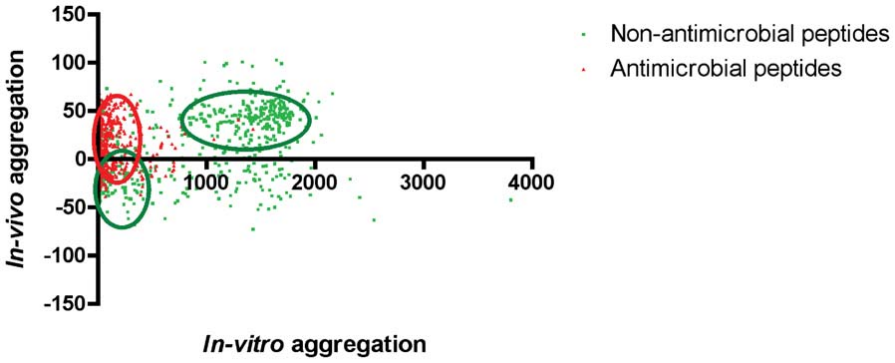
*In vivo* and *in vitro* aggregation propensity plot for AMPs (red triangles) and non-AMPs (green squares). *In vitro* aggregation propensity was computed using the TANGO software [26]. *In vivo* aggregation propensity was computed using AGGRESCAN software (The Na4vSS values computed represent the average aggregation propensity over the entire sequence divided by the number of residues and multiplied by 100) [27].

If TANGO and AGGRESCAN values are plotted together for AMPs and non-AMPs we can observe an interesting pattern (Figure 3). Whereas AMPs present high *in vivo* and very low *in vitro* aggregation propensity, non-AMPs can be divided in two main groups, presenting either high or low values for both descriptors. These results suggest that AMPs may minimize its aggregation in solution but promote aggregation in a more hydrophobic environment (i.e. the bacteria cell membrane). On the contrary, non-AMPs exhibit a dispersed aggregation pattern, probably related to their diverse biological functions. Thus, the results suggest that peptide's aggregation behaviour could be useful for classification purposes.

Finally, the hydrophobicity analysis (Figure 1H) shows that both the means and the variances differ significantly between AMPs and non-AMPs (p-value <0.0001). AMPs need to insert, partially or totally, into the membrane hydrophobic core in order to destabilize the bilayer and/or promote the cell depolarization [29]. Thus, a higher mean hydrophobicity value is expected for AMPs.

### Prediction of antimicrobial peptides by physicochemical properties

Using the physicochemical properties described above, an ANN system has been constructed in order to classify peptides in two groups: AMPs and non-AMPs. We have used the CAMP peptide database [17] to build the positive dataset and the Uniprot database to construct the negative dataset as described in the *Methods* section. After subtracting peptides containing non-standard amino acids the two datasets together contained 2148 peptides, 1157 AMPs and 991 non-AMPs. The ANN system (see the *Methods* section for further details) was evaluated using a training dataset containing 1074 peptides. The validation and testing datasets were populated each one with 537 peptides.

**Figure 4:**
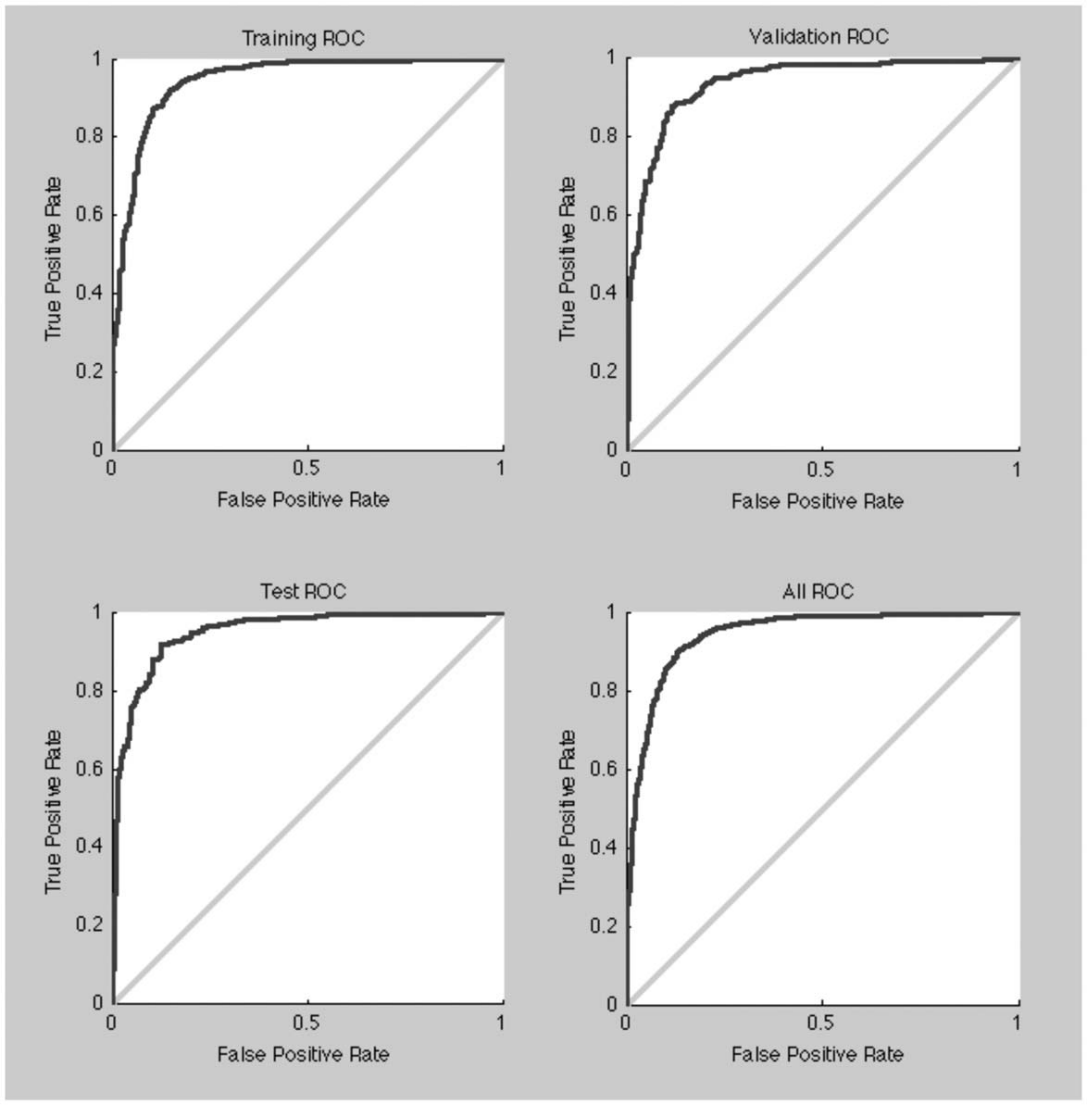
ROC curves for the training, validation, testing and global datasets showing the overall performance of the method described.

The receiver-operating curves (ROC) obtained (Figure 4) show that the method is able to correctly classify peptides in the two groups considered. The overall accuracy of the method was 90%, as shown in the corresponding confusion plot (Supporting Information, Figure S1) and is similar in both the validation and testing datasets, meaning that there was no over fitting. The use of 50 hidden neurons was optimum for the system designed. Less than 20 neurons consistently reduced the overall accuracy (<85%) and more than 50 nodes did not improve the result but might promote over convergence and a loss of model generality.

QSAR-based ANN methods described [19], using a set of 44 descriptors, obtained accuracy values near 80%. Our system can achieve a similar or even better accuracy using only 8 parameters. In any case, the comparison of accuracy values between the two methodologies has to be taken with caution, as different databases have been used. As the QSAR-based methods use two and three-dimensional structure estimations and similar descriptors for pI and hydrophobicity, we conclude that peptide aggregation could be of crucial importance in our classification system. Moreover, we have observed that structure and aggregation parameters have the most impact when deleted in the ANN analysis (data not shown).

A support vector machine (SVM) approach has also been tested in order to classify antimicrobial and non-antimicrobial peptides. In this case, we have only obtained a 75% correct classification using a 5-degree polynomial kernel (Supporting Information, Figure S2). Thus, in our system, a SVM approach has been found to be less accurate than the ANN approach. It is possible that the high amount of data and the reduced space dimensionality could favour ANNs over SVMs.

### Prediction of antimicrobial peptide potency by physicochemical properties

**Figure 5:**
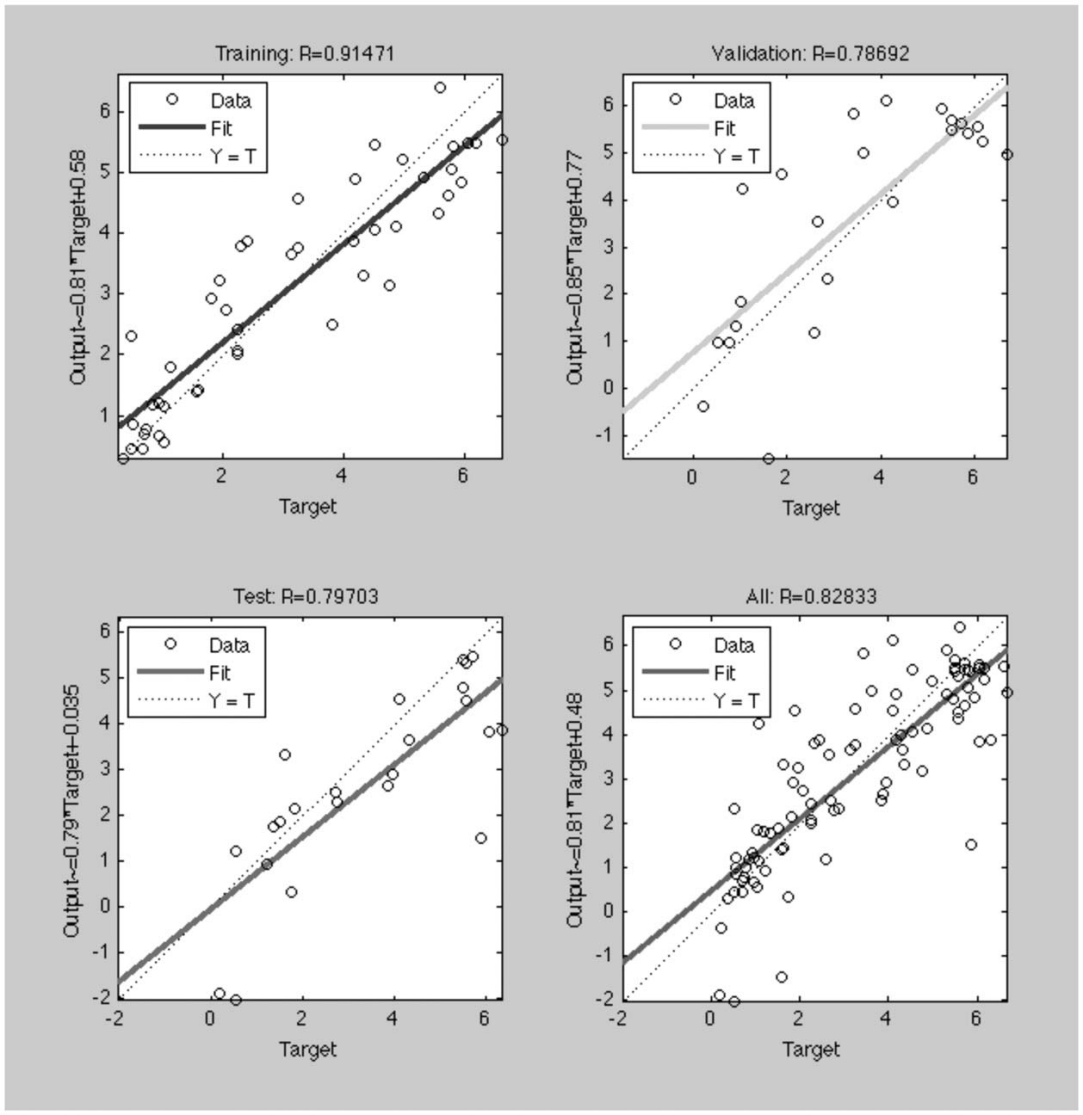
Regression model for the training, validation, testing and global datasets used in the antimicrobial activity prediction for the CAMEL peptide database. See the *Methods* section for a complete description of the methodology used.

To go a step further in this study we have tested our model for antimicrobial activity prediction using two independent and non-redundant data sets. We have used the data published by Cherkasov et al. [16] where the antimicrobial activity of different CAMEL variants was experimentally determined. The dataset employed is homogeneous, as all peptides were assayed in the same conditions and thus is of great value to test the parameters used in this study. From the 101 peptides described, we have used an ANN to compute the antimicrobial activity. The results obtained (Figure 5) show that our descriptors are closely correlated with antimicrobial activity (r^2^ = 0.72, q^2^ = 0.65).

**Figure 6:**
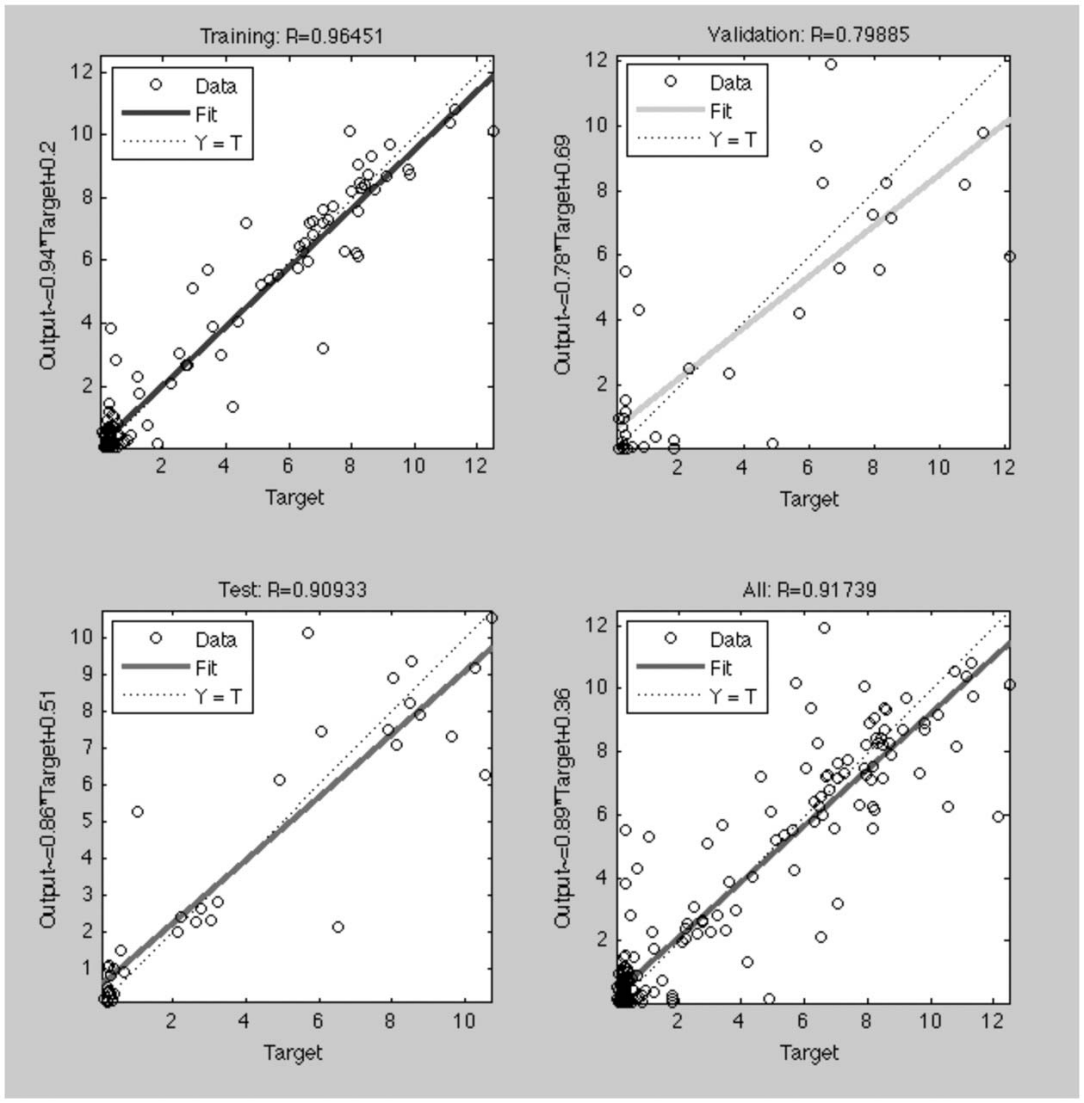
Regression model for the training, validation, testing and global datasets used in the antimicrobial activity prediction for the RANDOM peptide database. See the *Methods* section for a complete description of the methodology used.

We have also inspected the database (named RANDOM database) described recently by Fjell et al. [19] that contents a set of 189 peptides randomly synthesized and experimentally tested, again in uniform conditions. The results confirm (Figure 6), once more, a good correlation between our model and the experimental data (r^2^ = 0.85, q^2^ = 0.72).

The successful results obtained both for peptide classification and antimicrobial activity prediction may suggest that our set of parameters is complete and can appropriately describe the antimicrobial mechanism of action of peptides.

### Model design for the antimicrobial peptides mechanism

**Figure 7:**
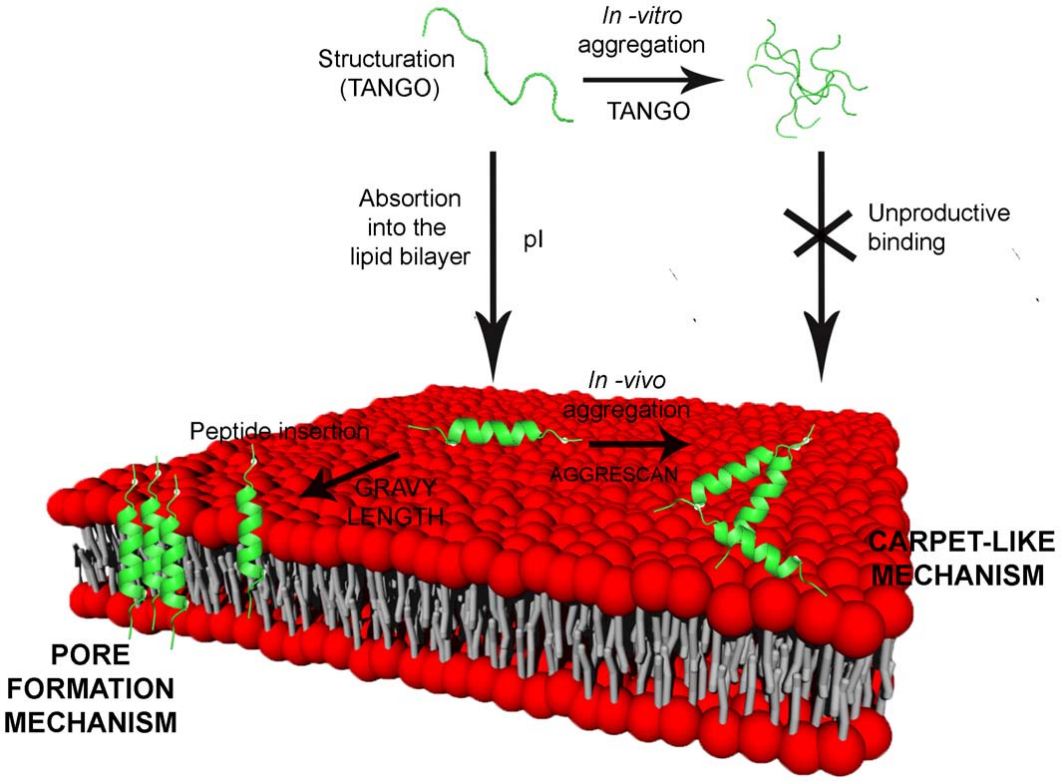
Representation of the model of action for AMPs. The figure depicts the main steps in the interaction and permeabilization of the bacterial membrane by AMPs. An arrow indicates each step. The peptide associated physicochemical parameters and the related prediction system used is also detailed. Although charge and hydrophobicity are also involved in peptide aggregation, only Tango and Aggrescan were included to this purpose as specific prediction systems.

As pointed before, a major interest of the present method lies in the ability to connect these physicochemical parameters with a global overview on the peptide mechanism of action (Figure 7). It is widely known that most of AMPs act mainly at the membrane level, destabilizing the bilayer structure by creating pores (toroidal or barrel-stave pores) or modifying its permeability by a mechanism known as “*carpet –like*” [22]. Both models promote the membrane depolarization and eventually the bacteria cell death.

The first step is the membrane binding, driven by the electrostatic interactions between the positive peptide charges and the negative charges located in the phospholipid polar heads [22]. It is known that, in certain conditions, some antimicrobial peptides can form aggregates in solution before they can interact with membranes [30], [31]. Most of these aggregates have been described to be inactive because they lack the ability to effectively insert totally or partially in the bilayer and thus are unable to promote the cell depolarization [32], [33]. For example, the aggregation of temporins prior to membrane interaction can avoid its activity [34], [35].

The following steps in the antimicrobial mechanism involve membrane destabilization and diverge depending on the particular action exerted by AMPs [22]. In a “*carpet-like*” mechanism, as observed in dermaseptin S and cecropin [36], formation of transmembrane structures is not necessary, but aggregation on the membrane surface and partial insertion is critical in order to promote destabilization and even bilayer micellization, as depicted in Figure 7. On the contrary, formation of toroidal pores, as observed for melittin [37], or barrel-stave pores, as observed for alamethicin [38], require a deep insertion in the membrane and a more ordered distribution of peptides across the bilayer.

In summary, the results presented here suggest that AMPs can be distinguished from non-AMPs by their physicochemical properties. We have also shown that peptide aggregation propensity must be included in the antimicrobial mechanism of action of peptides in order to correctly describe (and predict) its antimicrobial capacity. In addition, we have demonstrated that our method is able to correlate sequence-derived physicochemical peptide properties with the antimicrobial activity and thus is a fairly good approach to a general model able to describe and predict antimicrobial activity.

## Methods

### Database selection

The CAMP database [17] was used to construct the positive data set, containing 1157 peptides, where 95% of the peptides ranged from 10 to 50 residues. The Uniprot database was used to build the negative data set selecting peptides ranging between 10 and 50 amino acids length and filtered by Uniref50 in order to avoid over representative sequence similarities. None of the 991 selected peptides were reported as antimicrobial and/or toxic.

The data described by Cherkasov et al. [16] on antimicrobial CAMEL peptides (named CAMEL database) was used to assess the antimicrobial activity prediction. This database is composed of 101 peptides with experimentally tested antimicrobial potency. The data described by Fjell et al. [19] (named RANDOM database) on different antimicrobial peptides was also independently used to test the method. This database is composed of 189 peptides after discarding inactive peptides.

### Parameter computation

*In vitro* aggregation and secondary structure prediction were accomplished by using the TANGO software [26]. Tango calculates the partition function of the conformational phase space assuming that every segment on the protein populates one state: random coil, β-turn, α-helix, α-helix aggregation and β-sheet aggregation. Therefore, TANGO software can predict aggregation in solution, taking into account only structural parameters determined by the peptide sequence.

*In vivo* aggregation was computed using AGGRESCAN, an algorithm based on an amino acid aggregation-propensity scale derived from *in vivo* experiments and on the assumption that short and specific sequence stretches modulate protein aggregation. The algorithm can actually predict the aggregation propensity of peptides in the presence of cell material [27].

The isoelectric point (pI) was computed using the Expasy reference values and the peptide hydrophobic mean character using the GRAVY scale (http://expasy.org).

To study the differences in these parameters between AMPs and non-AMPs a two-tailed unpaired t-test analysis with a confidence interval of 95% has been used. All the parameters computed are considered to be independent as a correlation lower than 0.9 is observed between them in all the databases described above.

### Neural network implementation

Artificial neural networks (ANN) were computed using the Matlab software (Natick, MA). To predict antimicrobial peptides, a two-layer feed-forward network with sigmoid hidden and output neurons has been used. The network was trained with scaled conjugate gradient backpropagation. Hidden layer was populated with 50 neurons using 1074 peptides in the training database and 537 peptides on validation and testing databases. Both, matrix confusion plots and receiver-operative characteristic (ROC) curves were plotted for training, validation and testing databases.

To predict the antimicrobial potency of CAMEL and RANDOM antimicrobial peptides, a two-layer feed-forward network with sigmoidal hidden neurons and linear output neurons was used. The network was trained with Levenberg-Marquardt backpropagation algorithm. Regression plots were computed for training, validation and testing databases. The results obtained were cross-validated using the leave-20%-out methodology (q^2^ values).

### Support vector machine implementation

Support vector machine (SVM) models were computed using Gist SVM (http://svm.sdsc.edu). A training database of 1611 peptides and a testing database, containing 537 peptides, have been used. As the model contains variables with a heterogeneous scale, the data was adjusted to give a 0 mean value and 1 variance value in order to enhance performance. A two norm soft margin has been used and different polynomial kernels were tested in order to increase accuracy.

## Supporting Information

Figure S1 Confusion plot for the training, validation, testing and global datasets showing the positive and negative true and false rates for the method described. A legend is displayed on the top in order to help visual inspection. Abbreviations include: TP (true positives), FP (false positives), FN (false negatives), TN (true negatives), PPV (positive predicting value), NPV (negative predicting value), TPR (true positive rate or sensitivity), TNR (true negative rate or specificity) and ACC (accuracy).
(TIF)

Figure S2 Accuracy plot as a function of the Support Vector Machine kernel. A training database of 1611 peptides and a testing database, containing 537 peptides, have been used. A two norm soft margin was used in order to increase accuracy.
(TIF)
