# Supplementary figures and images for: Connecting Peptide Physicochemical and Antimicrobial Properties by a Rational Prediction Model
Source: PLoS One. 2011 Feb 9;6(2):e16968. doi: 10.1371/journal.pone.0016968 (PMC3036733; doi:10.1371/journal.pone.0016968)

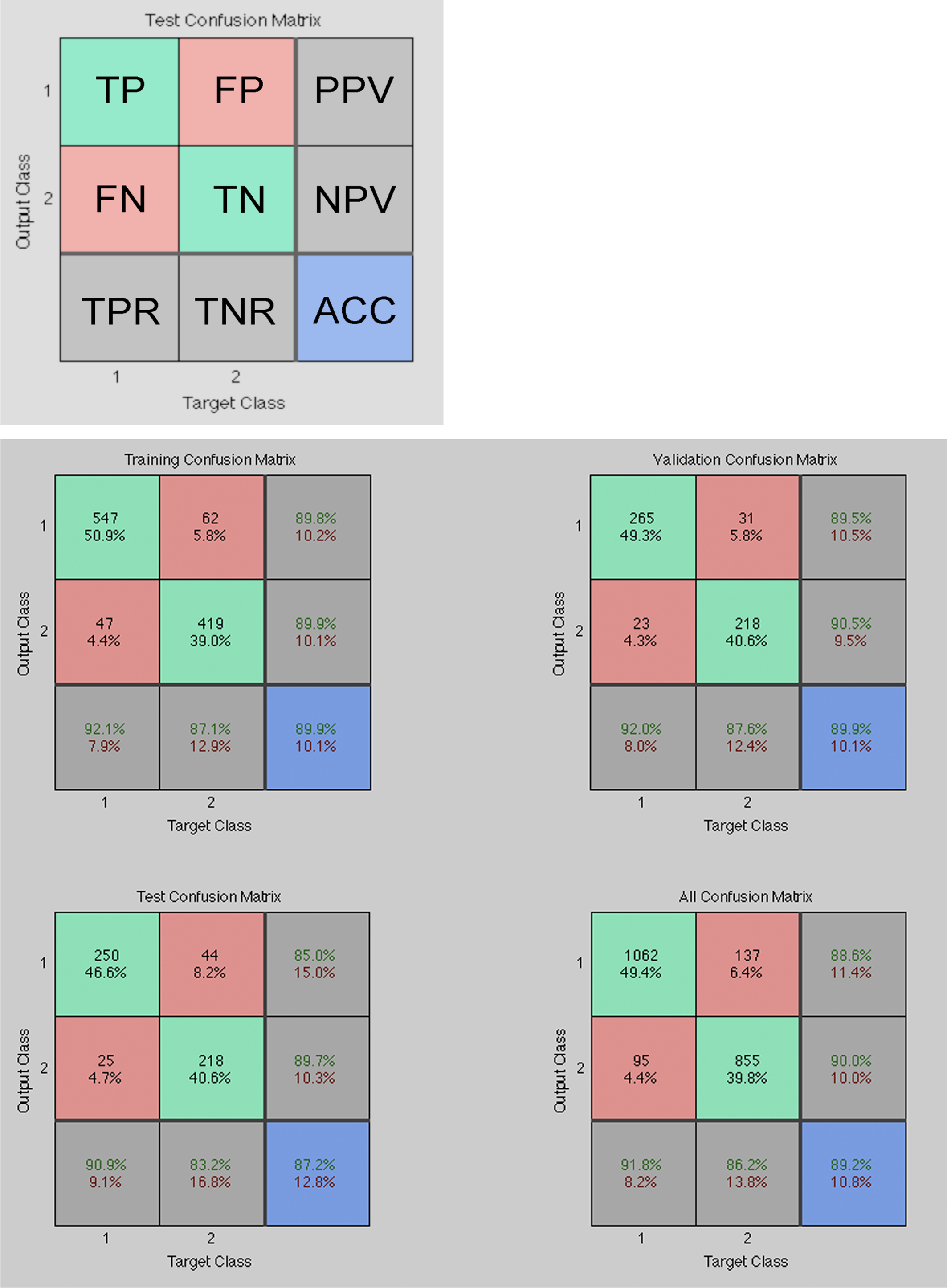

Supplement: Figure S1 — Confusion plot for the training, validation, testing and global datasets showing the positive and negative true and false rates for the method described. A legend is displayed on the top in order to help visual inspection. Abbreviations include: TP (true positives), FP (false positives), FN (false negatives), TN (true negatives), PPV (positive predicting value), NPV (negative predicting value), TPR (true positive rate or sensitivity), TNR (true negative rate or specificity) and ACC (accuracy). (TIF) [file pone.0016968.s001.tif]
